# Effects and Mechanism of Berberine on the Dexamethasone-Induced Injury of Human Tendon Cells

**DOI:** 10.1155/2020/8832218

**Published:** 2020-11-07

**Authors:** Shangjun Fu, Zongyun He, Yongfeng Tang, Bo Lan

**Affiliations:** Hand and Foot Surgery, Yiwu Central Hospital, Yiwu, 322000 Zhejiang, China

## Abstract

**Objective:**

To investigate the effects of berberine (Berb) on dexamethasone- (Dex-) induced injury of human tendon cells and its potential mechanism.

**Methods:**

CCK-8 assay was used to explore the appropriate concentration of Dex-induced injury of tendon cells and the doses of Berb attenuates Dex cytotoxicity; cell wound healing assay was used to detect the effects (*P* < 0.05) of Berb and Dex on the migration ability of tendon cells; flow cytometry was used to measure cell apoptosis; DCF DA fluorescent probe was used to measure the ROS activity of cells. Western blotting was used to detect the expression of phenotype related factors including smooth muscle actin *α* (SMA-*α*), type I collagen (Col I), col III, apoptosis-related factors, caspase-3, cleaved caspase-3, caspase-9, cleaved caspase-9, and PI3K/AKT.

**Results:**

CCK-8 assay showed that 1–100 *μ*M Dex significantly inhibited the proliferation of tendon cells in a concentration-dependent manner (*P* < 0.05), where the inhibitory effect of 100 *μ*M Dex was most significant (*P* < 0.005), and the pretreatment of 150, 200 *μ*M Berb could reverse those inhibitions (all *P* < 0.05). Compared with the control group, Dex significantly inhibited cell migration (*P* < 0.05), while Berb pretreatment could enhance cell migration (*P* < 0.05). Flow cytometry and ROS assay showed that Dex could induce apoptosis and oxidative stress response of tendon cells (all *P* < 0.05), while Berb could reverse those responses (*P* < 0.05). Western blot showed that Dex could inhibit the expression of the col I and III as well as *α*-SMA (all *P* < 0.05) and enhance the expression of apoptosis-related factors including cleaved caspase-3 and cleaved caspase-9 (all *P* < 0.05). Besides, Dex could also inhibit the activation of the PI3K/AKT signaling pathway (all *P* < 0.05), thus affecting cell function, while Berb treatment significantly reversed the expression of those above proteins (all *P* < 0.05).

**Conclusion:**

Berb attenuated DEX induced reduction of proliferation and migration, oxidative stress, and apoptosis of tendon cells by activating the PI3K/AKT signaling pathway and regulated the expression of phenotype related biomarkers in tendon cells. However, further studies are still needed to clarify the protective effects of Berb in vivo.

## 1. Introduction

Tendinopathy includes all pain related to the tendon injury, and activity-related pain is its main clinical symptom [1]. It has been estimated that tendinopathy accounts for 30–50% of musculoskeletal diseases and can often affect the shoulder, elbow joint, patella, and Achilles tendon function [2, 3]. Although physical methods, such as local hot compress, massage, and infrared physiotherapy, are often used clinically to treat tendon injuries, the effect is often not satisfactory [4]. At present, the treatment of tendinopathy usually relies on limited scientific evidences [1], and clinical experts often disagree about the use of some of these methods. Among those, the application of glucocorticoids, such as the commonly used dexamethasone (dexamethasone, Dex), is still very controversial and questionable [5]. Previous in vitro studies have shown that exposure to glucocorticoids has many negative effects on tendon cells [6]. For example, it can cause a decrease in the viability and proliferation of tendon cells and can reduce the level of total collagen (especially collagen 1 and proteoglycan) [7, 8]. The above effects can destroy the structure of the tendon, which is specifically shown as a decrease in the mechanical properties of the tendon [6] and can eventually lead to the occurrence of adverse events such as tendon rupture. Even given the side effects of glucocorticoid application on tendon cells, it is undeniable that hormones within glucocorticoid play a significant role when it comes to anti-inflammation. Therefore, in the research of clinical treatment of tendinopathy, it is necessary to find an adjuvant drug that can reduce glucocorticoid-induced tendon cell injury.

Berberine (Berb), also known as berberine hydrochloride, has been used as a traditional medicine in China for thousands of years [9]. It has a variety of protective biological functions such as antioxidant, anti-inflammatory, and antiapoptosis [10, 11]. Currently, a large number of clinical trials have confirmed the effectiveness of Berb on nonalcoholic fatty liver, irritable bowel syndrome, insulin resistance, and other diseases [12]. Therefore, this study intends to establish a Dex-induced human tendon cell injury model and to observe whether Berb can exert a protective effect on tendon cell injury, and to further study its mechanism, ultimately aiming to reduce the adverse effects of glucocorticoid treatment of tendinopathy.

## 2. Materials and Methods

### 2.1. Materials and Reagents

Dulbecco's modified eagle medium (DMEM), fetal bovine serum, penicillin-streptomycin liquid, phosphate buffer, and trypsin-EDTA digestion solution were all purchased from Hyclone, USA. Cell Counting Kit-8 kit was purchased from Dojindo, Japan, and Annexin V-FITC/PI apoptosis kit was purchased from Thermofisher, USA. Both Berb and Dex were purchased from Sigma-Aldrich, USA. RIPA protein lysate, antibody diluent, Tween-20, and ECL chemiluminescence reagents were all purchased from Shanghai Biyuntian Biotechnology Co., Ltd. Primary antibodies for smooth muscle actin *α* (smooth muscle actin *α*, *α*-SMA), type I collagen (Col I), col III, caspase-3, cleaved caspase-3, caspase-9, cleaved caspase-9, p-PI3K, PI3K, p-AKT, AKT, and *β*-actin were all purchased from Abcam, USA. The reactive oxygen species (ROS) detection kit was purchased from Shanghai Biyuntian Biotechnology Co., Ltd.

### 2.2. Method

#### 2.2.1. Human Tenocyte Culture

This research protocol has been approved by the Human Research Ethics Committee of our hospital. Primary human tenocytes were prepared from the patellar tendons of healthy volunteers for culture by following [8]. After the volunteers signed the informed consent, when the anterior cruciate ligament was reconstructed with autologous bone-patellar tendon-bone transplantation, a tendon tissue block (2 × 2 × 3 mm) was cut from the central 1/3 of the healthy patellar tendon for primary cell culture. The tissue block was washed with a phosphate buffer solution containing 1% penicillin-streptomycin liquid (100 U/mL penicillin and 100 *μ*g/mL streptomycin) first before cutting the tissue block into 1 mm^3^ size under aseptic conditions. The tissue was then digested with trypsin-EDTA. After 5 minutes of digestion, the digested tissue pieces were then transferred to a 35 mm petri dish containing DMEM with 10% fetal bovine serum and 1% penicillin-streptomycin liquid and cultured in an incubator at 37°C with 5% CO_2_. The medium was replaced twice a week. After observing the tendon cells and cell fusion in the tendon tissue under the microscope, the tendon fibroblasts were then digested and cultured in the culture flask with the inoculation density of 10^5^ cells per flask under the same culture conditions for subculture. Subsequent experiments were conducted using the cells that were less than 5 passages.

#### 2.2.2. Establishment of Dex-Induced Tendon Cell Injury Model

Dex was dissolved in absolute ethanol to prepare a storage solution with a concentration of 10 mM and stored in a refrigerator at −20°C. Before processing the tenocytes, the stock solution was diluted with DMEM supplemented with 10% fetal bovine serum and 1% penicillin-streptomycin liquid to a final concentration of 1, 10, and 100 *μ*M, respectively. Tendon cells were treated with the above concentrations for 48 h to induce cell damage, and the Dex concentrations that can significantly inhibit cell proliferation activity were selected for follow-up studies.

#### 2.2.3. Detection of Cell Proliferation Activity by CCK-8

*(1) Test the Effect of Dex on the Proliferation Activity of Tenocytes*. For specific operations, see Method 1.2.2. Tenocytes were seeded in a 96-well plate at a density of 5 × 10^3^ cells/well, cultured for 24 h under standard conditions and then treated with Dex at different concentrations (1, 10, and 100 *μ*M) for 48 h. After treatment, 10 *μ*L CCK-8 was added, and the cells were put in the incubator again and continued in culture for 2 h. After that, a spectrophotometer was used to measure the absorbance value (absorbance, A) at 450 nm wavelength to calculate the relative cell proliferation activity. Each group had 5 replicates. The value measured from the DMEM was used as the background value to eliminate the interference of the medium itself on the absorbance value. Relative cell proliferation activity = (A treatment group-A background group)/(A control group-A background group).

*(2) Detect the Effect of Berb on the Proliferation Activity of Dex-Induced Tenocytes*. First, different concentrations of Berb (0, 25, 50, 100, 150, 200, and 300 *μ*M) were used to pretreat tendon cells for 24 h, and then Dex was used to induce tendon cell injury for 48 h. For subsequent operations, see Method (1). The appropriate concentration that can effectively reduce the cell damage caused by Dex was selected for follow-up research.

#### 2.2.4. Evaluation of Cell Migration Ability by the Scratch Test

Tenocytes were seeded in a six-well plate at a density of 2 × 10^5^ cells/well, and they were divided into the control group (Control), Dex group, 150 Berb + Dex group (150 *μ*M Berb pretreatment), and 200 Berb + Dex group (200 *μ*M Berb pretreatment). After the inoculated cells were cultured for 24 h, the 150 Berb + Dex group and 200 Berb + Dex group were pretreated with 150 and 200 *μ*M Berb for 24 h, respectively, and then treated with Dex for 48 h. After the treatment, the cells were digested and reseeded in a six-well plate at a density of 1 × 10^6^ cells/well. After 24 h of culture, a 200 *μ*L sterile pipette tip was used to draw 3 parallel lines that were vertical to the plate. After washing the floating cells with PBS, normal DMEM was added, and pictures were then taken under an inverted microscope. After imaging, the cells were then returned to the incubator to continue culturing for 24 h, and then the pictures were taken again. Image J was then used to measure the width of the scratch (W), and the relative migration capacity of cells was calculated with formula (W-0 h-W-24 h)/W-0 h.

#### 2.2.5. Detection of ROS Level

The DCF-DA fluorescent probe was used to detect the ROS activity of cells. The cells were seeded in a 48-well plate at a density of 1 × 10^4^ cells/well and processed as method 2.2.4 for specific operations. The cells were washed twice with PBS, and 200 *μ*L of 25 *μ*M DCF-DA was then added, and the cells were then put back in the incubator for 30 min. After the incubation, the cells were washed twice with PBS again, and then the relative fluorescence intensity of the sample was measured with the Synergy Mx multifunction microplate detector at the excitation and emission wavelengths of 485 and 528 nm, respectively.

#### 2.2.6. Detection of Apoptosis by Flow Cytometry

Annexin V-FITC/PI double staining was used to detect cell apoptosis. First, the cells were inoculated in a six-well plate. After each group of cells was treated accordingly (see Method 2.2.4 for details), the cells were then digested with trypsin-EDTA digestion solution and centrifuged at 800 g for 5 min to obtain the cell pellet. 500 *μ*L PBS was then added to wash the cell pellet before centrifuging again to collect the pellet. After repeating the washing 3 times, 500 *μ*L of binding buffer was added to the cell pellet to resuspend the cells. After that, 15 *μ*L of Annexin V-FITC and PI were added in sequence, and cells were then incubated at room temperature for 30 min in the dark. After the incubation, flow cytometry was then used for apoptosis detection.

#### 2.2.7. Expression of Protein Detected by the Western Blotting

*(1) Effects of Berb on the Protein Expression of Dex-Induced Tendon Cells*. RIPA Lysis Buffer was used to lyse the treated cells on ice, cells were then centrifuged at 12,000 g/min for 20 min to take the cell lysate supernatant, and the BCA protein concentration determination kit was then used to determine the protein concentration. 30 *μ*g protein sample was then taken and loaded on the SDS-PAGE gel, and the protein was separated by electrophoresis and transferred to the PVDF membrane. The membrane was blocked at room temperature for 1 h and then inoculated at 4°C with primary antibodies (SMA, col I, col III, caspase-3, cleaved caspase-3, caspase-9, cleaved caspase-9, p-PI3K, PI3K, P-AKT, AKT, and *β*-actin) overnight. After washing the membrane the next day, the membrane was then incubated in the corresponding secondary antibody for 1 h at room temperature. After washing the membrane again, enhanced chemiluminescence reagent was added for protein exposure and development, and Photoshop software was then used for protein grayscale analysis.

*(2) Test the Effects of PI3K Inhibitors and Verify the Expression of PI3K/AKT Signaling Pathway Proteins*. The cells were divided into 5 groups, namely, the control group (control), Dex group, LY294002 group, Berb + Dex group, and LY294002 + Berb + Dex group. After the corresponding treatment, the specific detection steps were conducted as shown in Method (1).

#### 2.2.8. Statistical Analysis

The experimental data were analyzed and processed by SPSS 18.0. Quantitative data were expressed as mean ± standard deviation. Under the same treatment condition, the difference between the groups was compared by one-way analysis of variance; the Dunnett *t*-test was used for the comparison between different groups and the control group. *P* ≤ 0.05 was used for the statistically significant test. All experiments in the study were repeated independently at least three times.

## 3. Results

### 3.1. The Effect of Dex and Berb Treatment on the Proliferation Activity of Tenocytes

**Figure 1:**
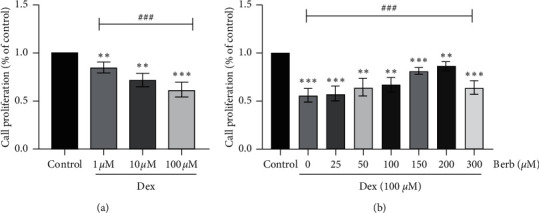
Effects of Dex and Berb on proliferation of tendon cells. (a) CCK-8 assay was used to detect the effects of various concentration of Dex (1, 10, and 100 *μ*M) on the proliferation of tendon cells; (b) CCK-8 assay was used to measure the effects of Dex on the proliferation of tendon cells pretreated with different concentrations of Berb (0, 25, 50, 100, 150, 200, and 300 *μ*M). *Note.* Compared with the control group, ^*∗∗*^*P* < 0.01 and ^*∗∗∗*^*P* < 0.001; compared within the treatment groups, ^###^*P* < 0.001.

As shown in Figure 1(a), with the increase of Dex treatment concentration, the proliferation activity of tenocytes decreased in a dose-dependent manner (*P* < 0.001), so the follow-up study chose 100 *μ*M Dex to treat tenocytes to induce cell damage. As shown in Figure 1(b), pretreatment of cells with different concentrations of Berb (0, 25, 50, 100, 150, 200, and 300 *μ*M) before exposure to 100 *μ*M Dex can reduce the damaging effect of Dex on cells in a dose-dependent manner (*P* < 0.001). The concentration of 150 and 200 *μ*M Berb treatment was most obvious, so 150 and 200 *μ*M were chosen as the Berb pretreatment concentration for follow-up research.

### 3.2. The Effect of Combined Treatment of Dex and Berb on the Migration Ability of Tenocytes

**Figure 2:**
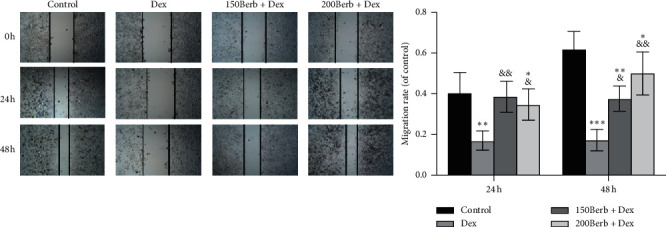
Berb attenuates the inhibitory effect of Dex on the migration of tendon cells. *Note.* Compared with the control group, ^*∗*^*P* < 0.05, ^*∗∗*^*P* < 0.01, and ^*∗∗∗*^*P* < 0.001; compared with Dex group, ^&^*P* < 0.05 and ^&&^*P* < 0.01.

As shown in Figure 2, compared with the control group, Dex treatment can significantly reduce the cell migration ability (all *P* < 0.05), while, compared with the Dex group, it can be seen that Berb pretreatment can promote the recovery of cell migration ability (all *P* < 0.05). Moreover, with the extension of the scratch culture time, the promotion effect becomes more obvious.

### 3.3. The Combined Effect of Dex and Berb on the Induction of Apoptosis of Tendon Cells

**Figure 3:**
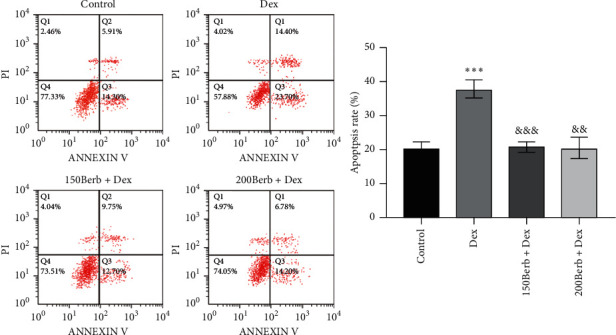
Berb attenuated Dex-induced apoptosis of tendon cells. *Note.* Compared with the control group, ^*∗∗∗*^*P* < 0.001; compared with Dex group, ^&&^*P* < 0.01, ^&&^*P* < 0.01, and ^&&&^*P* < 0.001.

As shown in Figure 3, flow cytometry showed that Dex can significantly induce apoptosis of tendon cells (*P* < 0.05), while Berb pretreatment can reduce the rate of apoptosis of tendon cells (all *P* < 0.05).

### 3.4. The Combined Treatment of Dex and Berb on the Induction of Oxidative Stress in Tendon Cells

**Figure 4:**
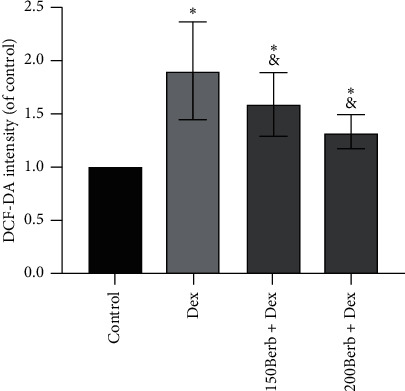
Effects of Dex and Berb on the oxidative stress response of tendon cells. *Note.* Compared with the control group, ^*∗*^*P* < 0.05; compared with the Dex group, ^&^*P* < 0.05.

As shown in Figure 4 (DCF-DA detection of ROS level), Dex can significantly induce oxidative stress in tendon cells, and ROS expression is significantly increased (*P* < 0.05), while Berb treatment can inhibit the production of ROS (all *P* < 0.05) indicating its inhibitory effects on the occurrence of oxidative stress.

### 3.5. The Effect of Dex and Berb on the Expression of Apoptosis Factors in Tendon Cells

**Figure 5:**
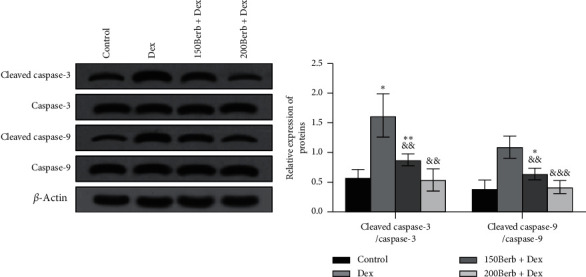
Berb inhibited Dex-induced expression of apoptosis related factors in tendon cells. *Note.* Compared with the control group, ^*∗*^*P* < 0.05, ^*∗∗*^*P* < 0.01, and ^*∗∗∗*^*P* < 0.001; compared with Dex group, ^&&^*P* < 0.01 and ^&&&^*P* < 0.001.

As shown in Figure 5, compared with the control group, Dex exposure can significantly increase the expression of cleaved caspase-3 and cleaved caspase-9 in cells (both *P* < 0.05) and induce the occurrence of apoptotic response, while Berb pretreatment can reverse the above changes (all *P* < 0.05) thus playing an antiapoptotic effect.

### 3.6. The Combined Effect of Dex and Berb on the Expression of Phenotype-Related Biomarkers in Tendon Cells

**Figure 6:**
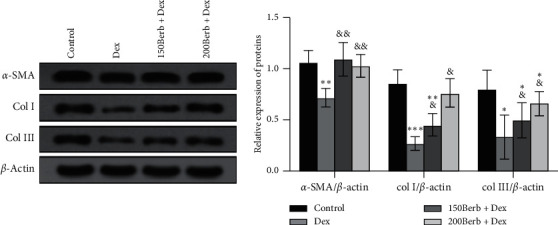
Effects of Dex and Berb on the expression of phenotype related biomarkers in tendon cells. *Note.* Compared with the control group, ^*∗*^*P* < 0.05, ^*∗∗*^*P* < 0.01, and ^*∗∗∗*^*P* < 0.001; compared with Dex group, ^&^*P* < 0.05 and ^&&^*P* < 0.01.

As shown in Figure 6, compared with the control group, Dex treatment can significantly inhibit the expression of *α*-SMA, col I, and col III (all *P* < 0.05), thereby destroying cell function, while Berb pretreatment promoted the expression of *α*-SMA, col I, and col III (all *P* < 0.05), suggesting that Berb can contribute to the functional recovery of damaged tendon cells.

### 3.7. The Combined Effect of Dex and Berb on the Activation of PI3K/AKT Signaling Pathway

**Figure 7:**
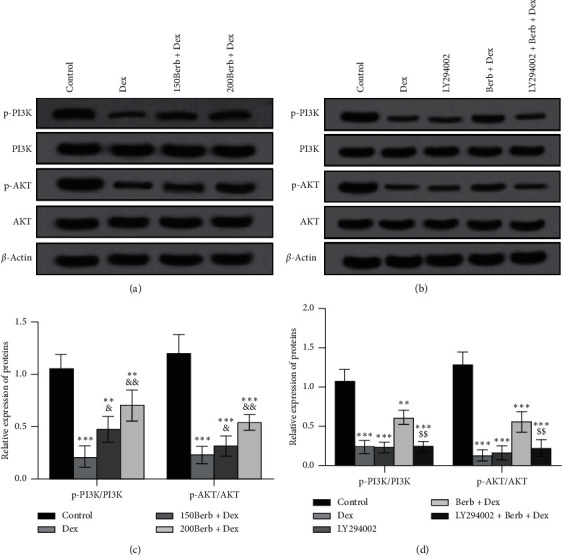
Berb treatment induced activation of PI3K/AKT signaling pathway in tendon cells. (a) Dex inhibited the activation of PI3K/AKT signaling pathway, which was reversed by Berb pretreatment; (b) PI3K inhibitor verified the activation of PI3K/AKT signaling pathway. *Note.* Compared with the control group, ^*∗*^*P* < 0.05, ^*∗∗*^*P* < 0.01, and ^*∗∗∗*^*P* < 0.001; compared with Dex group, ^&^*P* < 0.05 and ^&&^*P* < 0.01; compared with Berb + Dex group, ^$$^*P* < 0.01.

As shown in Figure 7, Dex exposure can inhibit the activation of the PI3K/AKT signaling pathway in tendon cells (all *P* < 0.05), while Berb pretreatment can reverse this change (all *P* < 0.05). The addition of PI3K inhibitor LY294002 further confirmed this effect.

## 4. Discussion

The healing of tendon injuries involves many complex processes, which could span stages of inflammation, regeneration, and remodeling [13]. In this process, tendon cells migrate to the repair site, proliferate actively, and are responsible for depositing a large amount of extracellular matrix in the tissue. The normal progress of the above cell activities is the basis of tendon healing [14]. Previous studies have found that while Dex is anti-inflammatory, it can cause damage to the function of tendon cells, which may hinder the regeneration process of tendon healing [15]. Berb is the main isoquinoline alkaloid component of many common medicinal plants (such as barberry, coptis, etc.), due to its multiple biological activities, including antidiarrheal, anticancer, antidiabetic, antihyperlipidemia, and heart protection, it has been widely used in traditional Chinese medicine [16]. Because of the bioprotective effect of Berb, this study aims to investigate whether Berb can reduce Dex-induced tendon cell damage and promote the recovery of cell function.

In this study, the proliferation and migration functions of tenocytes were studied by detecting cell proliferation activity, finding the appropriate concentration of Dex-induced damage and the intervention concentration of Berb that can inhibit Dex damage. The CCK-8 experiment found that when 100 *μ*M Dex is given to tenocytes, the proliferation activity of the cells can be significantly inhibited. This result is consistent with the results of Wong et al. [8]; that is, Dex can significantly reduce the viability of tenocytes and inhibit cell proliferation in a dose-dependent manner. Besides, this study also found that Berb can dose-dependently mitigate the reduction of cell proliferation induced by Dex in the concentration ranging from 25 to 200 *μ*M. Furthermore, in the study on the change of the migration ability of damaged tenocytes, we found that Dex can significantly reduce the migration ability of cells; however, when cells were treated with Berb in advance and then exposed to Dex, the damaged migration ability of the cells can be significantly reversed, and, with the extension of the treatment time, the effect of improving cell migration ability is further enhanced. The above cell function experiments suggest that Berb can play a cytoprotective effect and reduce the damage of proliferation and migration ability caused by Dex.

This study also observed the inducing effect of Dex on the apoptosis and oxidation of tendon cells and the corresponding protective effect of Berb. By measuring cell apoptosis via flow cytometry, we found that Dex can significantly induce cell apoptosis. However, when cells were pretreated with 150 and 200 *μ*M Berb, the rate of apoptosis was significantly reduced. We also confirmed the occurrence of apoptosis by detecting the protein expression levels of apoptosis-related factors including caspase-3 and caspase-9. It is known that caspase-3 can be activated by caspase-9 cleavage. The activation of the caspase signal and its downstream apoptotic response pathway is an important feature of the apoptotic cascade [17]. Cleaved caspase-3 and caspase-9 are important biomarkers for the progression of apoptosis. Besides the results of flow cytometry for the detection of the apoptosis response, this study also found that Berb pretreatment can significantly reduce the increase in the expression of apoptotic proteins in tendon cells induced by Dex, thereby further verifying the antiapoptotic effect of Berb. Previously, Min et al. used Dex to induce human tendon cell injury. When studying the mechanism of vitamin D on tendon cells, they found that vitamin D can reduce the ROS produced by Dex [18]. In the oxidation reaction experiment, by detecting the strength of DCF-DA, which indicates the ROS level, it was found that Dex can increase the ROS level of tendon cells and cause cell oxidative damage, while the administration of Berb can reverse the occurrence of oxidation reaction, thereby exerting a protective effect. The above experiments suggest that Berb can not only promote the recovery of tenocyte function but also reduce cell damage by inhibiting Dex-induced oxidation and apoptosis.

Collagen (col) is one of the important constituents of tendon cells, accounting for 70% of the dry weight of the tendon [19]. In the normal tendons, col I make up for about 90% of the collagen, and the rest is col III [20]. Col I usually form a tissue bundle by forming parallelly arranged fibers, while col III is normally confined to the inner membrane around the bundle formed by col I. When the injury occurs, tenocytes can produce col I and III to help tendon healing [21]. Studies have shown that, in human tendon tissues such as the anterior cruciate ligament, up to 50% of the cells contain *α*-SMA, which plays an important role in maintaining the structure of the extracellular matrix [22]. Given the evidence mentioned above, col I, col III, and *α*-SMA were selected as the biological markers of tendon cell phenotype in this study, and the changes of their corresponding protein expressions were also measured to indicate the repair level of tendon cells. We found that the expression of these three proteins in the damaged cells induced by Dex was significantly reduced; however, treatment with Berb could reverse this change, suggesting the function of Berb in maintaining the phenotype of tenocytes and promoting its repairing.

It is known that AKT, as a mechanical conversion molecule, can affect the development and homeostasis of various musculoskeletal tissues, including muscle (stretch), cartilage (compression), and bone (shear force) [23]. Previous studies have confirmed that the PI3K/AKT signaling pathway plays an important role in the process of tendon differentiation and tendon formation [23]. Therefore, in the study of the mechanism of Berb's protective effect, this study measured the activation level of the PI3K/AKT signaling pathway. The results showed that Dex can inhibit the activation of PI3K/AKT, while Berb pretreatment can promote the activation of this pathway. To confirm the relationship between PI3K/AKT signal transduction, Dex, and Berb, we used the PI3K inhibitor LY294002 in the combined action of Dex and Berb. We found that, based on Berb's activation of the PI3K/AKT signaling pathway, the addition of PI3K inhibitors can again inhibit the activation of this pathway suggesting that both Dex and Berb can inhibit and activate the PI3K/AKT signaling pathway, respectively.

## 5. Conclusion

This study found that pretreatment with Berb can regulate the activation of the PI3K/AKT signaling pathway and reverse the decrease in the proliferation and migration of tenocytes caused by Dex-induced injury. The pretreatment of Berb also decreased the occurrence of apoptosis and oxidative stress caused by Dex, as well as restoring the expression of biological markers related to the phenotype of tenocytes. The above results suggest that Berb has a cytoprotective effect, but further in vivo studies are still needed to reveal the beneficial effects of Berb on tendons.

## Data Availability

The data used to support the findings of this study are available from the corresponding author upon request.
